# IGF IIRα-triggered pathological manifestations in the heart aggravate renal inflammation in STZ-induced type-I diabetes rats

**DOI:** 10.18632/aging.203244

**Published:** 2021-07-07

**Authors:** Henry Cherng-Han Lin, Catherine Reena Paul, Chia-Hua Kuo, Yung-Hsien Chang, William Shao-Tsu Chen, Tsung-Jung Ho, Cecilia Hsuan Day, Vijaya Padma Viswanadha, Yuhsin Tsai, Chih-Yang Huang

**Affiliations:** 1 Graduate Institute of Chinese Medicine, China Medical University, Taichung404, Taiwan; 2 Cardiovascular and Mitochondrial Related Disease Research Center, Hualien Tzu Chi Hospital, Buddhist Tzu Chi Medical Foundation, Hualien97004, Taiwan; 3 Laboratory of Exercise Biochemistry, University of Taipei, Taipei11153, Taiwan; 4 Department of Chinese Medicine, China Medical University Hospital, China Medical University, Taichung404, Taiwan; 5 Department of Psychiatry, Tzu Chi General Hospital, Hualien97004, Taiwan; 6 School of Medicine, Tzu Chi University, Hualien97004, Taiwan; 7 Department of Chinese Medicine, Hualien Tzu Chi Hospital, Buddhist Tzu Chi Medical Foundation, Tzu Chi University, Hualien97004, Taiwan; 8 Integration Center of Traditional Chinese and Modern Medicine, Hualien Tzu Chi Hospital, Buddhist Tzu Chi Medical Foundation, Hualien97004, Taiwan; 9 School of Post-Baccalaureate Chinese Medicine, College of Medicine, Tzu Chi University, Hualien97004, Taiwan; 10 Department of Nursing, MeiHo University, Pingtung912, Taiwan; 11 Department of Biotechnology, Bharathiar University, Coimbatore641046, India; 12 Graduate Institute of Chinese Medicine, China Medical University, Taichung41354, Taiwan; 13 Graduate Institute of Biomedical Sciences, China Medical University, Taichung404, Taiwan; 14 Center of General Education, Buddhist Tzu Chi Medical Foundation, Tzu Chi University of Science and Technology, Hualien970, Taiwan; 15 Department of Medical Research, China Medical University Hospital, China Medical University, Taichung404, Taiwan; 16 Department of Biotechnology, Asia University, Taichung413, Taiwan

**Keywords:** diabetes, renal fibrosis, cardiac stress, embryonic gene, cardiorenal syndrome

## Abstract

Pathological manifestations in either heart or kidney impact the function of the other and form the basis for the development of cardiorenal syndrome. However, the mechanism or factors involved in such scenario are not completely elucidated. In our study, to find the correlation between late fetal gene expression in diabetic hearts and their influence on diabetic nephropathy, we created a rat model with cardiac specific overexpression of IGF-IIRα, which is an alternative splicing variant of IGFIIR, expressed in pathological hearts. In this study, transgenic rats over expressing cardiac specific IGF-IIRα and non-transgenic animal models established in SD rats were administered with single dose of streptozotocin (STZ, 55 mg/Kg) to induce Type I diabetes. The correlation between IGF-IIRα and kidney damages were further determined based on their intensity of damage in the kidneys. The results show that cardiac specific overexpression of IGF-IIRα elevates the diabetes associated inflammation and morphological changes in the kidneys. The diabetic transgenic rats showed advancement in the pathological features such a renal tubular damage, collagen accumulation and enhancement in STAT3 associated mechanism of renal fibrosis. The results therefore show that that IGF-IIRα expression in the heart during pathological condition may worsen symptoms of diabetic nephropathy in rats.

## INTRODUCTION

The global incidence and prevalence of diabetes mellitus (DM) is on increase for the last 20 years [1]. Cardiovascular complications like coronary artery, cerebrovascular and peripheral artery diseases increase the morbidity and mortality rates among diabetic patients. Diabetes causes deterioration in the function of heart and the kidney and may lead to cardiorenal syndrome (CRS) in which deleterious effect of either of the organ affects the function of the other and further amplifies the pathological effects [2]. However, the underlying mechanism for deterioration and the factors that initiate the trigger and maintain the interaction is not understood yet [2]. According to the previous studies, CRS may affect through two main known pathways. The first one is the pro-inflammatory reaction which positively links with the severity of the disease [3, 4] and the second one is the inappropriate upregulation of the Renin Angiotensin System, a notable event in both chronic kidney disease and chronic heart failure [3] And recent studies show that cardiovascular participation occurs in each stage of chronic kidney disease and further major cardiac actions represent almost 50% mortality in cardiac kidney disease patients [5]. Understanding the mechanism of CRS has remained difficult as it has many complex physiological, biochemical, and hormonal abnormalities. In this study, in order to further understand the role of pathological expression of late stage fetal gene in the heart on diabetes associated cardiorenal syndrome and in exacerbating diabetes associated renal damages have been studied.

Hypertrophic remodeling is an evident process in the response to pathological manifestations such as diabetes. The response of heart to hemodynamic or metabolic stress often leads to the activation of fetal genes and reduction in the post-natal gene expression. Long-term pathological hypertrophy of the heart is considered as the major clinical predictor of heart failure. Although the factors that drive hypertrophic effects contributes to effective adaptations to stress, unbalances in their expression affects cardiac contractility and myocardial energetics leading to functional deterioration. Activation of the “fetal gene program” is considered to be the interlayer between pathological cardiac remodeling and the pathogenesis of heart failure [8]. Remarkably, recovery from heart failure and improvement in ventricular function upon treatment with beta-blockers or with ventricular assist devices are often associated with downregulation in the expression of cardiac fetal genes [6–8].

Our previous researches demonstrate that signaling mechanism involving insulin-like growth factor II receptor (IGFIIR), a fetal gene, is key in the pathological progression of hypertrophy [9, 10]. Reactivation of IGFIIR signaling in the heart during stresses leads to cardiac hypertrophy and heart failure. IGFIIR is a type I transmembrane glycoprotein whose activation and its cell surface expression in cardiomyocytes leads to cardiomyocyte apoptosis. Our previous studies show that IGFIIR is expressed during late stage cardiac events and an alternative splicing variant of IGFIIR named IGF-IIRα has been identified in pathological heart [11, 12]. Elevated expression of IGF-IIRα during pathological conditions like diabetes and high salt worsens the cardiac performance and increases tissue damages [13–15]. A transgenic IGFIIRα overexpression rat model was created with cardiac specific Myh6 promoter. The IGF-IIRα transgenic mice have been associated with ventricular hypertrophy and cardiomyocyte damages [13–16]. IGFIIR has been suggested to enhance the activation of TGF-β levels in patients with chronic heart failure by cleaving latent TGF-β which could correlate with increasing risk for chronic kidney disease. However, the renal effects of cardiac specific IGF-IIRα expression is not yet established. In this study we evaluated the effect of cardiac IGF-IIRα expression on diabetes associated renal inflammation and deterioration of tissue homeostasis. The results will help in understanding the cardiac markers for potential targets.

## RESULTS

### Cardiac specific IGF–IIRα overexpression worsens DM associated kidney weight change

**Figure 1:**
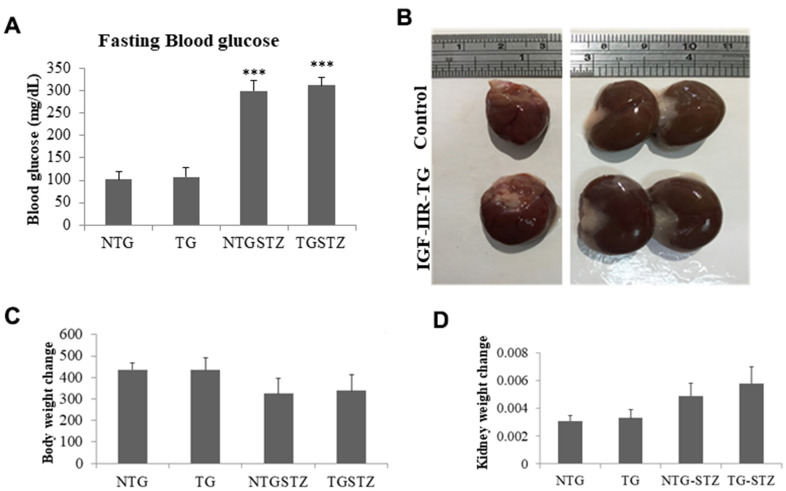
**Effects of cardiac specific IGFIIRα overexpressing DM rats on kidney weight.** (**A**) Fasting blood glucose levels in Non-transgenic rats (n=6, NTG), transgenic (n=6, TG), NTG-streptozotocin induced diabetes model (n=6, NTGSTZ) and TG streptozotocin induced diabetes model (n=6, TGSTZ). (**B**) Cardiac specific IGF-IIRα overexpression causes hypertrophy of heart correlated with enlarged kidneys. (**C**) Changes in body weight and (**D**) Changes in kidney weight among NTG, TG, NTGSTZ, TGSTZ. *** *p*<0.001 indicates significance when compared to NTG group.

As shown in Figure 1A, a single dose of STZ induced diabetes mellitus with significant (p<0.001) increase in blood glucose level however, cardiac specific over expression of IGF–IIRα did not influence the hyperglycemic levels in the DM rats. Cardiac IGF–IIRα in DM rats causes hypertrophy of the heart that was also correlated with enlargement in kidney size (Figure 1B). Meanwhile, the rats induced with STZ showed notable reduction in the body weight however there was no effect of IGF–IIRα overexpression on the diabetes associated bodyweight change (Figure 1C). DM in IGF–IIRα overexpressing TG rats showed increase in the kidney size as reflected in the kidney weight/tibia length ratio (Figure 1D). The results show that IGF–IIRα overexpression in heart does not affect the general effects of DM such as blood glucose and bodyweight change in the rats; however it inflicts a strong influence on kidney.

### Effects of cardiac specific IGF–IIRα overexpression on the serum biochemical markers

**Figure 2:**
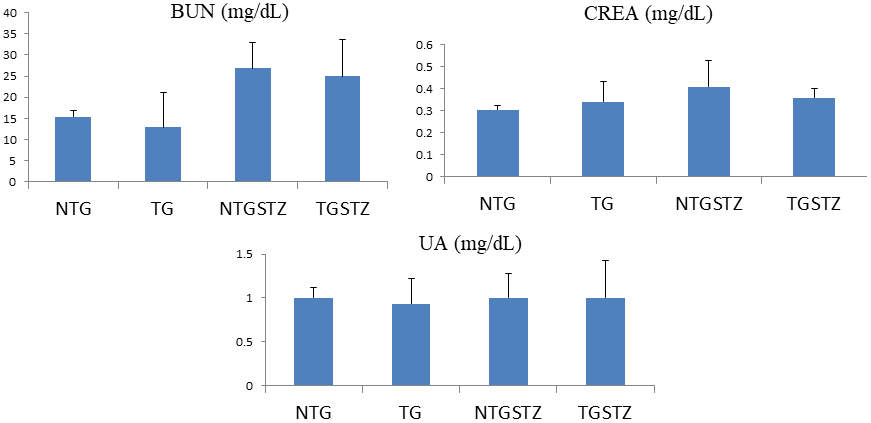
**Effects of cardiac specific IGFIIRα overexpressing DM rats on Blood serum.** Blood serum analysis (n=6) shows difference in blood urea nitrogen (BUN), creatinine (CREA) and uric acid (UA)levels between Non-transgenic rats (NTG), transgenic (TG), NTG-streptozotocin induced diabetes model (NTGSTZ), TG streptozotocin induced diabetes model (TGSTZ).

The serum lipid profile showed high serum levels of kidney tissue markers such as BUN, CREA, and UA in DM TG and Non-TG rats. However, there was no change attributed to the IGF–IIRα overexpression. The results show that IGF–IIRα overexpression in hearts doesn’t show any effects on the biochemical markers in DM Non-TG rats and TG rats (Figure 2).

### Effects of cardiac specific IGF–IIRα overexpression on the histological analysis

**Figure 3:**
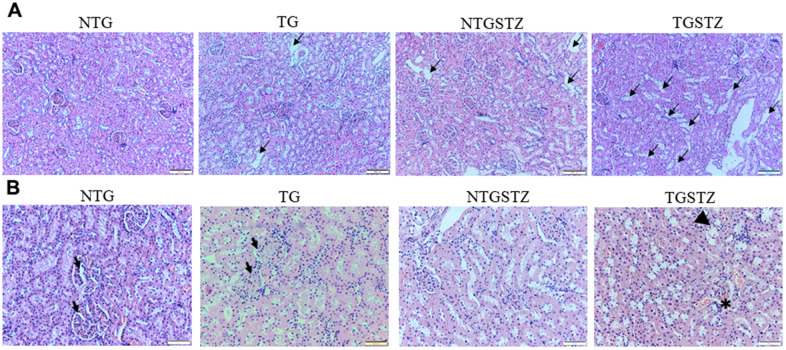
**Effects of cardiac specific IGFIIRα overexpressing DM rats in renal cellular architecture.** (**A**) H and E staining shows changes in renal cellular architecture in Non-transgenic rats (NTG), transgenic (TG), NTG-streptozotocin induced diabetes model (NTGSTZ), TG streptozotocin induced diabetes model (TGSTZ). TG rats show renal tubular damages (arrows) and STZ induced DM in TGSTZ elevates the damages. (**B**) Glomeruli of TG rats showed a slight hypertrophy and mesangial expansion (Arrow). NTGSTZ groups showed contraction of glomeruli. TGSTZ rats show higher degree of glomeruli infiltration (*) and damages in addition TGSTZ rats show atrophy and degeneration of renal tubules (arrow head). Scale bar represent 100 μm at 40x magnification.

**Figure 4:**
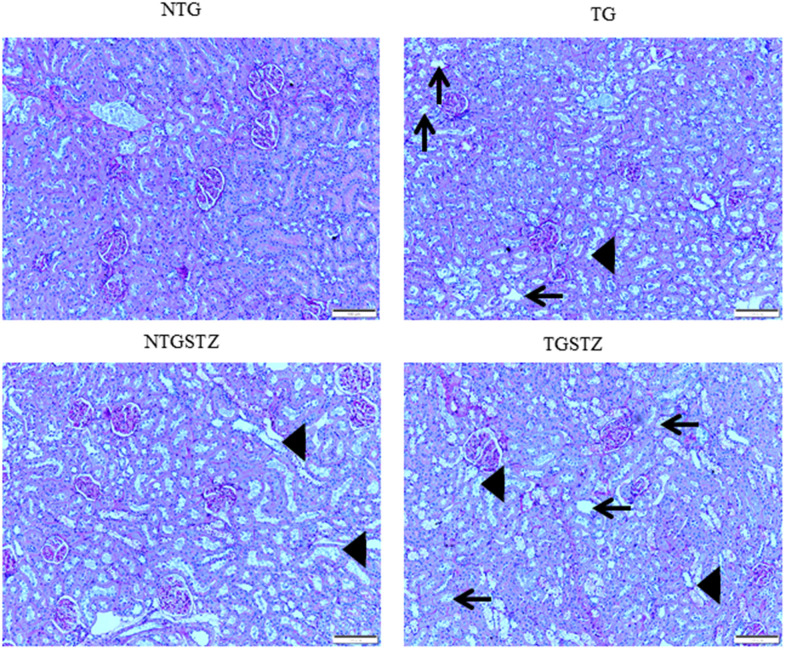
**PAS staining to show changes in the tubular structures.** PAS staining show differences between Non-transgenic rats (NTG), transgenic (TG), NTG-streptozotocin induced diabetes model (NTGSTZ), TG streptozotocin induced diabetes model (TGSTZ). STZ challenge triggered vacuolization (arrow) and degeneration of renal tubular epithelium NTGSTZ. TG rats that generally showed tubular dilation (arrow head) also showed higher levels of STZ induced vacuolization and degeneration. Scale bar represent 100 μm at 40 x magnification.

**Figure 5:**
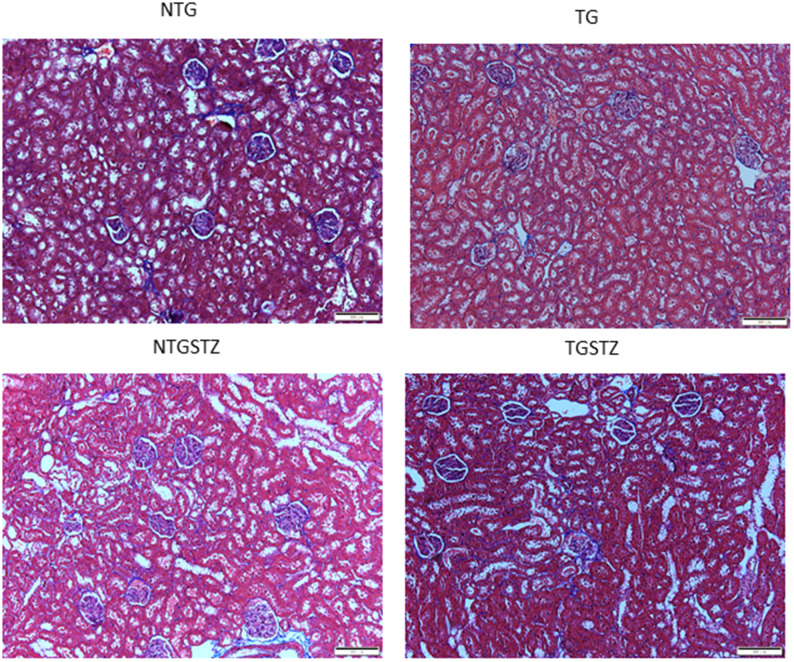
**Effects of cardiac specific IGFIIRα overexpressing DM rats on interstitial collagen.** Masson’s trichrome staining (n=3) show difference in collagen accumulation in Non-transgenic rats (NTG), transgenic (TG), NTG-streptozotocin induced diabetes model (NTGSTZ), TG streptozotocin induced diabetes model (TGSTZ). NTGSTZ induced DM rats show collagen accumulation (blue stain). TG rats show elevated interstitial collagen accumulation compared to NTG and NTGSTZ rats. Scale bar represent 100 μm at 40 x magnification.

After STZ induction the kidney tissues were examined for the histological changes using H&E staining, PAS staining and Masson’s trichrome staining. The H&E staining showed changes in renal cellular architecture (Figure 3). TG rats showed renal tubular damages and STZ induced DM elevated the tubular damages (Figure 3A). And also the glomeruli structure of TG rats showed a slight hypertrophy and mesangial expansion (Figure 3B). Meanwhile, STZ induced Non-TG groups showed contraction of glomeruli. TG rats showed higher degree of glomeruli infiltration and damages in addition TG DM rats showed hypertrophy and degeneration of renal tubules. PAS staining showed changes in the tubular structures (Figure 4). STZ challenge triggers vacuolization and degeneration of renal tubular epithelium in Non–TG rats. But in the case of TG rats, that generally showed tubular dilation, and TG DM rats, higher levels of vacuolization and degeneration was observed. According to Masson’s trichrome staining to determine renal fibrosis (Figure 5), STZ induced DM rats showed collagen accumulation and TG DM rats showed elevated interstitial collagen accumulation when compared to control and Non-TG DM rats.

### Effects of cardiac specific IGF –IIRα overexpression in heart modulates fibrosis associated inflammation markers in kidney

**Figure 6:**
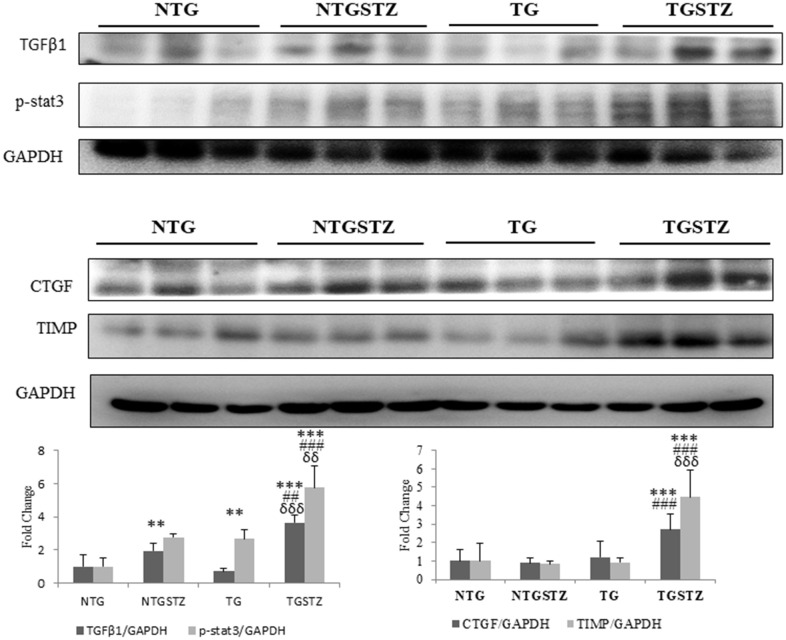
**Changes in diabetic nephropathy associated Inflammation mediators.** Western blotting analysis (n=3) on DM associated inflammatory cytokines showed differences in the levels of TGFβ1, p-stat3, CTGF and TIMP that are associated with fibrosis in kidney, among Non-transgenic rats (NTG), transgenic (TG), NTG-streptozotocin induced diabetes model (NTGSTZ), TG streptozotocin induced diabetes model (TGSTZ). **<*p*0.01 and *** *p*<0.001 indicate significance when compared to NTG group. ##<*p*0.01 and ### *p*<0.001 indicate significance when compared to NTGSTZ group and δδ<*p*0.01 and δδδ *p*<0.001 indicate significance when compared to TG group.

The western blotting assay was used to assess the effect of IGF –IIRα overexpression in heart modulates inflammation markers in kidney tissues. The inflammation associated proteins showed increase in the levels of p-Stat3 in the STZ groups that seems to be associated with fibrosis in kidney (Figure 6). Pro-fibrotic marker TGFβ1 that activates epithelial–to mesenchymal transition has been associated with chronic kidney diseases by activating pro fibrotic gene expression. STZ induced DM in Non-TG rats upregulated the levels of TGFβ1 and significantly (*p*<0.01) increased its downstream transcription activator p-stat3; in the TG rats the DM caused an exaggerated upregulation of TGFβ1 and p-Stat3 (*p*<0.01) (Figure 6). Similarly, CTGF associated with fibroblast activation and TIMP, an inhibitor of fibrinolytic enzymes was increased significantly (*p*<0.001) in the TG DM rats. The results therefore show that DM associated stress is intensified upon cardio specific overexpression of the late stage embryonic protein IGF –IIRα.

### Effects of cardiac specific cardiac IGF–IIRα overexpression in heart modulates survival and apoptosis markers in kidney tissues

**Figure 7:**
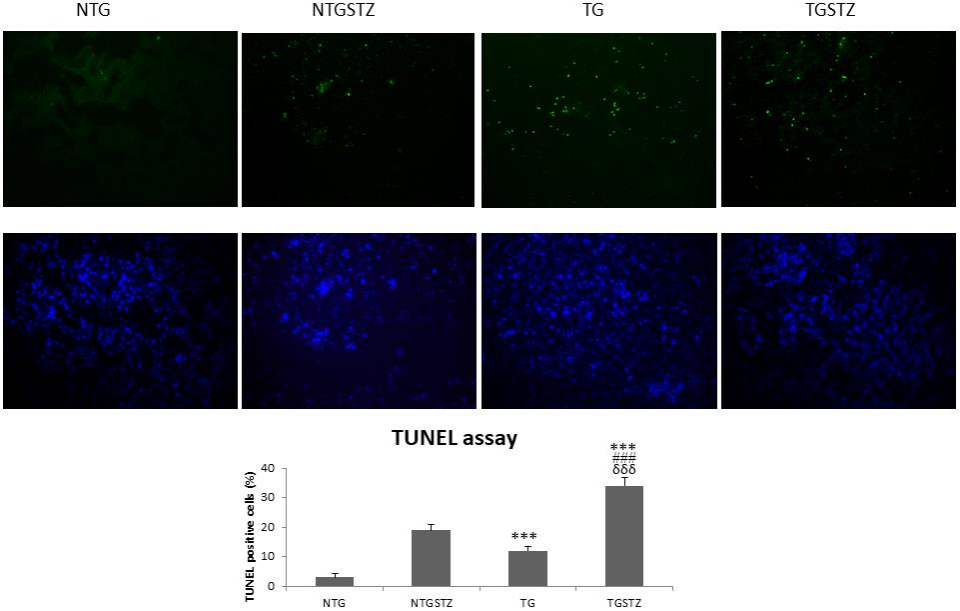
**Effects of cardiac specific IGFIIRα overexpressing DM rats on apoptosis.** STZ induced DM rats show TUNEL positive nuclei (green). IGF2R overexpression in hearts increases the apoptosis rate in kidney (n=3). Scale bar represent 100 μm at 40 x magnification. *** *p*<0.001 indicates significance when compared to NTG group. ### *p*<0.001 indicates significance when compared to NTGSTZ group and δδδ *p*<0.001 indicates significance when compared to TG group.

TUNEL assay to find the number of apoptotic nuclei also show that TG DM rats undergo significantly (*p*<0.001) higher apoptosis levels (Figure 7). The results therefore reveal that expression of the late stage embryonic gene IGF–IIRα in the heart may cause kidney cell to further succumb to the pathogenicity.

## DISCUSSION

Significant inventions from our laboratory show that IGF-IIRα signaling controls cardiac hypertrophy that leads to heart failure [13–17] here we describe on the association of novel IGF-IIRα shows a key controlling role in cardiac structure in over expressed TG rats that affects. In this study, we demonstrated IGF-IIRα overexpression in heart intensifies renal damage in end stage kidney failure condition. It is known that, chronic heart failure develops from an insufficiency in cardiac output which in turn interrupts renal perfusion and may lead to vasomotor nephropathy. Our data from the morphological findings states that, cardiac IGF-IIRα overexpression is tangled in renal remodeling and renal function decline. Cardiac-IGF-IIRα overexpressed rats revealed visible changes in kidney weight, kidney weight –body weight ratio. Based on the well-established staining methods to show any abnormal renal cellular architecture, we found a slight hypertrophy and mesangial expansion. H and E staining showed the difference in the glomerular abnormalities between DM TG and Non-TG rats, glomeruli showed a higher degree of infiltration in the DM TG rats and in addition hypertrophy and degeneration in renal tubules was visible. According to PAS staining TG rats showed tubular dilation that was aggravated in DM rats with notable vacuolization and degeneration in DM TG rats. And also the Masson’s trichrome staining showed elevated interstitial collagen accumulation in DM TG rats when compared with control and Non TG DM rats. The above mentioned differences in cellular architecture reveal that cardiac specific overexpression of IGF-IIRα has notable effects in Kidney. In addition to the cellular architecture studies, Western blotting assay show the elevated tissue expression of TGFβ1 in DM TG rats as compared to Non-TG DM rats.

Diabetic nephropathy (DN) is one among the serious chronic complications of type 1 diabetes. Patients with type 1 diabetes develop tissue abnormalities and break down in homeostasis in blood flow and glomeruli vascular permeability that are indicated by abnormal levels of albumin in the urine, a condition referred as microalbuminuria [18]. The production of nitric oxide decreases in the renal capillaries eventually causing increasing sensitivity to angiotensin II [19]. The Glomerular filtration rate (GFR) decreases over a period of 10-20 years that highly varies among individuals and in Patients with microalbuminuria leading to development of hypertension along the way which leads to end-Stage renal disease [20].

Recent findings show that signal transducer and activator of transcription 3 (STAT3) is one of the vital signaling pathways in the development of diabetic nephropathy [21, 22]. JAK/STAT is a crucial signaling cascade in the pathogenesis of diabetic nephropathy [23]. It is significant in the regulation of fundamental cellular processes such as growth, differentiation, immunity and survival. Its eccentric activity is also linked to carcinogenesis and tumor progression and has been of great interest as a therapeutic target. Evidence specifies that activation of the JAK/STAT pathway in renal glomerular mesangial cells activates excessive cell proliferation as well as unlimited production of TGF-β1, collagen IV, and fibronectin, contributing to the glomerulosclerosis in diabetic nephropathy [24–27]. In addition, activated STAT3 in tubular cells has been stated to be important in chronic kidney disease. Experimental evidence found from a murine kidney injury model using unilateral ureteral obstruction links STAT3 with amplified deposition of extracellular matrix proteins, thereby driving the development of renal fibrosis [21, 28]. Our present results show that the proteins involved in the STAT3 mediated renal fibrosis mechanism are highly elevated in the DM TG rats with cardiac specific IGF-IIRα overexpression. This suggests that cardiac damages and associated late stage embryonic gene expression could inflict changes in the cardio-renal axis and causes increased stress in the kidney cells that ultimately leads to cellular damages and renal fibrosis.

Coronary artery disease is seen commonly among patients with chronic kidney disease and a large number of non-hemodynamic factors have been attributed to the development of cardiomyopathy in chronic kidney disease patients [29]. Hyperphosphatemia associated with kidney disorders is linked to increased blood pressure [30] and is also known to induce cardiac hypertrophy and further affect the viability of cardiac cells due to excessive autophagy [31, 32]. Chronic kidney disease is also associated with excess angiotensin II accumulation in the heart that eventually promotes cardiac hypertrophy and fibrosis resulting in dysfunction and arrhythmias [33]. The activation of the renin-angiotensin system may lead to increase in serum aldosterone and the levels of TGF β- a causative factor for cardiac fibrosis [34–36].

However, in the recent years more focus has been made on the cardiac factors that are involved in the chronic kidney disease. Exosomes and non-coding RNAs released by heart cells have been reported to be the mediators of cardiac hypertrophy in *in vitro* models [37]. These cardiac secretory factors may also potentially affect other distal tissues like the kidney.

In conclusion our results suggest that renal damages in diabetic condition are due to multifaceted pathological events which include direct effects of hyperglycemia on kidney and also due to pathological manifestation in the heart. Therefore, treatment approaches for diabetic nephropathy should consider strategies to target cardiac late stage proteins in addition to the conventional methods.

## MATERIALS AND METHODS

### Animal models

Five weeks old male SPRAGUE-DAWLEY (SD) were purchased from BioLASCO (Taipei Taiwan) and the TG rats were purchased from National animal laboratory Animal center, Taiwan [14]. The rats were maintained at 24 ± 2° C temperature and humidity was maintained around 55 ± 10% with light and dark cycle every 12h. A standard laboratory diet (Lab Diet 5001; PMI Nutrition International Inc., Brentwood, MO, USA) and drinking water ad libitum. Rats were divided into four groups after one week of adaptation. The groups are Control group (n=6), TG group (n=6), STZ induced control group (STZ, n=6) and the 4^th^ group was TG group with STZ induced (n=6). The control and TG rats were given PBS and the STZ induced group rats received STZ (55mg /kg body weight) when the rats are around eight weeks. This study followed the principles specified by laboratory animal care (NIH publication) and was approved by the Institutional Animal Care and Use Committee of China Medical University, Taiwan.

### Blood biochemical analysis

In this study, all blood biochemical tests were gathered and measured by China Medical University Hospital, what's more, the accompanying parameters were broke down to investigate the capacity of Kidney: BUN (Blood Urea Nitrogen), CREA (Creatinine), UA (Uric Acid), study showed the protein profile in serum.

### Protein extraction and western blotting

Cortex region from kidney was homogenized in a lysis buffer (0.05M of pH 7.4 Tris-HCl, 1 mM EDTA, 0.15 M NaCl, 1% NP-40, and 0.25% deoxycholic acid) at a ratio of 100mg tissue 1mL buffer. The homogenates were stored overnight in -80° C and centrifuged at 13,000 rpm for 40 min protein in the supernatants were collected and again stored at -80° C for further analysis. Lowry protein assay were used to determine the protein concentration of kidney tissue extracts. The protein samples were separated using western blot techniques using 8-12 % SDS polyacrylamide gel electrophoresis under 60V for 150min. The separated proteins were later transferred to PVDF membranes (GE Healthcare Limited, Buckinghamshire, UK) under 60V for 90 mins. The transferred proteins in PVDF membrane were blocked using % bovine serum albumin (BSA) for 1 hour and later intermixed with primary antibodies using tris-buffered saline. Finally the blots were intermixed with horseradish peroxidase-labeled secondary antibodies and using LAS-3000 (GE Healthcare UK Limited, Buckinghamshire, UK) pictures were taken.

### Hematoxylin and eosin staining

The attached rat kidney sections were cut into 0.2 μm thick slices and deparaffinized using xylene by immersing the slides. And rehydrated using (100%-60%) alcohol. And stained with hematoxylin and eosin (H&E) and photomicrographs were taken using Zeiss Axiophot microscopes.

### PAS staining

The attached rat kidney tissues were cut into 0.2 μm thick slices and fixed with 10% formaldehyde for approximately 24 h followed by dehydration and made transparent, and fixed in paraffin. After dewaxing, the embedded tissue sections were stained with 1% periodic acid solution, and Schiff’s solution was added to the incubator at 37° C. After treatment, the sections were sealed with glue and photographed under a fluorescence inverted microscope.

### Masson’s trichrome staining

The rat kidney tissues were cut into 0.2 μm thick slices and fixed with 10% formalin and treated with chains of alcohol gradient like 75% 85%, 90%, and 100% liquor, 5 min each) and fixed in paraffin wax. The kidney tissues were deparaffinized using xylene by immersing the slides. Then immersed in preheated Bouin’s fluid for specified duration and later rinsed with tap water. Later the sections were stained with the following solutions like Weigert’s iron hematoxylin solution, Biebrich scarlet-acid fuchsin, phosphotungstic-phosphomolybdic acid solution, aniline blue solution, and 1% acetic acid solution with alternating washing procedure. The slides were dehydrated in 95% ethanol and lastly washed with ethanol and xylene and fixed in synthetic resin.

### Immunohistochemistry

The attached rat kidney tissue section were cut into (5 μm) thick slices and incubated with anti- TGF β1 (1:200) primary antibody. Immunohistochemistry analysis was performed according to a proven protocol.

### Statistics

Results are conveyed as means ± SE (Standard Error) of three independent results. Evaluations between each groups were made with (ANOVA) one-way analysis of variance followed by Tukey test using GraphPad Prism version 5 (GraphPad Software Inc., La Jolla, CA, USA).
